# Chocolate flavanols and skin photoprotection: a parallel, double-blind, randomized clinical trial

**DOI:** 10.1186/1475-2891-13-66

**Published:** 2014-06-27

**Authors:** Jaime Andres Mogollon, Catherine Boivin, Simone Lemieux, Claudine Blanchet, Joël Claveau, Sylvie Dodin

**Affiliations:** 1 St. François d’Assise Hospital, Centre hospitalier universitaire de Québec (CHUQ), Quebec, QC, Canada; 2 Melanoma and Skin Cancer Clinic, Dermatology Division, Hôtel-Dieu Hospital, CHUQ, Quebec, QC, Canada; 3 Department of Food Sciences and Nutrition, Institute of Nutrition and Functional Foods (INAF), Université Laval, Quebec, QC, Canada; 4 Department of Obstetrics and Gynecology, Research Centre, St. François d’Assise Hospital (CHUQ), Université Laval, Quebec, QC G1L 3 L5, Canada

**Keywords:** Skin photoprotection, Flavanols, Chocolate, Cocoa, Minimal erythema dose

## Abstract

**Background:**

Solar ultraviolet (UV) radiation has deleterious effects on the skin, including sunburn, photoaging and cancer. Chocolate flavanols are naturally-occurring antioxidant and anti-inflammatory molecules that could play a role in preventing cutaneous UV damage. We investigated the influence of 12-week high-flavanol chocolate (HFC) consumption on skin sensitivity to UV radiation, measured by minimal erythema dose (MED). We also evaluated skin elasticity and hydration.

**Methods:**

In this 2-group, parallel, double-blind, randomized controlled trial, 74 women aged 20–65 years and Fitzpatrick skin phototypes I or II were recruited from the general community in Quebec City, for randomization to either HFC (n = 33) or low-flavanol chocolate (LFC) (n = 41). A blocked randomisation (4), considering date of entry, skin type and age as factors, generated a sequentially-numbered allocation list. Study participants and research assistants were blinded. Totally, 30 g of chocolate were consumed daily for 12 weeks, followed by a 3-week washout period. MED was assessed at baseline and at 6, 9, 12 and 15 weeks. Main outcome was changes in MED at week 12.

**Results:**

33 participants in the HFC group and 41 in the LFC group were analyzed with 15 weeks of follow-up. Both groups showed similarly-increased MED at 12 weeks (HFC: 0.0252 ± 0.1099 J/cm^2^ [mean ± standard deviation (SD)]; LFC: 0.0151 ± 0.1118; mean difference (MD): 0.0100 J/cm^2^; 95% confidence interval (CI): -0.0417 to 0.0618). However, after 3-week washout, the HFC group presented decreased MED (-0.0248 ± 0.1145) whereas no effect was seen in the LFC group (0.0168 ± 0.1698) (MD: -0.0417; 95% CI: -0.1106 to 0.0272). Net temple elasticity increased slightly but significantly by 0.09 ± 0.12 mm in the HFC group at 12 weeks compared to 0.02 ± 0.12 mm in the LFC group (MD: 0.06; 95% CI: 0.01 to 0.12 ). No significant adverse events were reported.

**Conclusion:**

Our study failed to demonstrate a statistically-significant protective effect of HFC vs. LFC consumption on skin sensitivity to UV radiation as measured by MED.

**Trial registration:**

ClinicalTrials.gov identifier: NCT01444625

## Introduction

Associated with morphological and physiological changes, aging of the skin is a complex process that reflects inevitable chronological aging accentuated by environmental factors. Although several genetic and environmental elements interplay, the most well-understood mechanism by which skin aging occurs is through chronic solar ultraviolet (UV) radiation, known as photoaging [1,2]. Indeed, chronic solar UV exposure has multiple damaging effects, such as wrinkling, dryness, dyspigmentation, epidermal thinning and increasing fragility. Changes, especially in elastic fibers of the dermis, result in loss of flexibility and tensile strength, with collagen shortfall, inducing skin stiffness [1]. UV radiation is one of the most ubiquitous carcinogens in our environment, and skin cancers represent one of the major consequences of excessive exposure. In addition, evidence is growing in support of the view that UV levels are rising because of stratospheric ozone depletion and climate change [3-6].

Various strategies are possible for skin support in defense against damaging environmental influences, particularly sun exposure. The World Health Organization recommends wearing protective c1othing, avoiding mid-day sun and applying sunscreen. Yet, studies have disclosed that on sunny days no more than 65-67% of Danish sunbathers use sunscreen at the beach, and only 46% of sunscreen users applied it all over their body [7]. In a Canadian study, 40% to 48% of individuals reported practising protective behaviours, such as applying sunscreen to the body, wearing protective clothing, seeking shade and avoiding the sun between 11 am and 4 pm [8].

The concept of photoprotection by dietary means is gaining momentum, and interest is growing in natural dietary polyphenols for the prevention of UV-induced damage [2,7,9]. Flavanols, a subclass of polyphenols, possess efficient antioxidant [10-14] and anti-inflammatory [15-20] properties. Recent but scarce data indicate that chronic ingestion of high-flavanol cocoa might be a promising approach to dietary photoprotection against UV light. [21-23] Furthermore, flavanol-rich cocoa intake has been shown to increase microcirculation in human skin [24]. Nutritional photoprotection with flavanol-rich chocolate is a promising area for research, but double-blind clinical trials are required to confirm experimental findings.

As flavanols exert antioxidant, anti-inflammatory and anti-DNA-damaging effects that may enhance the skin’s microcirculation, we hypothesized that chronic flavanol-rich chocolate consumption may represent an effective strategy to protect against harmful UV radiation.

Our study’s primary objective was to investigate the impact of 12-week high-flavanol chocolate (HFC) consumption vs. LFC on skin sensitivity to UV radiation, measured by minimal erythema dose (MED). Its secondary objectives were to assess the effects of HFC vs. LFC on mechanical skin elasticity by cutometer and hydration by corneometer.

## Methodology

### Research design

We undertook a 2-group, parallel, double-blind, randomized clinical trial (RCT) with a 3-week washout period.

### Ethics statement

The protocol and consent form for this study were approved by the institutional health science research ethics committee of Université Laval, Quebec. Appointments were scheduled for eligible women, where the risks and benefits of their possible participation were reviewed in detail. The informed consent form was read and signed by them before study inclusion.

### Study participants

Between July 2011 and March 2012, we enrolled non-smoking healthy women aged 20 to 65 years who had normal skin types I or II, as described by Fitzpatrick [25].

We excluded patients with one or more of the following conditions: pregnancy or breast- feeding, photosensitivity, history of skin cancer, photosensitizing medication, sunbed tanning or sunbathing in preceding 3 months, planned sunbed tanning or sunbathing during the study period, supplements of any kind (fish oil, coenzyme Q-10, garlic, lycopene, beta-carotene, etc.), except for medically-prescribed supplements or natural health products, consumption of ≥2 alcoholic drinks per day, allergy or intolerance to nuts or chocolate, body mass index (BMI) >35, hormone replacement therapy (HRT) or hormonal contraception in the preceding 6 months before the pre-randomization visit, or planned HRT or hormonal contraception during the study period. Women with systolic blood pressure ≥160 mmHg, diastolic blood pressure ≥100 mmHg, or treated with antihypertensive medication(s) were also excluded.

### Recruitment and randomization

Women were recruited from the general population of Quebec City through websites, email, newspapers, radio-television advertising, and flyers posted in clinics. Potential study participants in the study contacted the study coordinator who explained the research project to them and verified inclusion and exclusion criteria.

### Allocating participants to trial groups

At the randomization visit, participants were randomly assigned to either HFC (experimental group) or low-flavanol chocolate (LFC, placebo group). The randomization schedule was prepared at the St-François d'Assise Research Centre statistics unit. A blocked randomisation (4) was computer-generated by a statistician who was not involved in the study. It was stratified according to skin type (I and II) and age (30–35 years; 36–49 years; 50–65 years). A first list of randomisation was generated according to an equal number of participants in each age and skin type stratum. After three months of recruitment, proportion of women with skin type 2 and age 50–65 were more prevalent than expected and a new independent list of randomisation was generated.

### Intervention

#### Daily chocolate intake (30 g)

Study participants consumed 1 chocolate square 3 times per day (30 g/day) for 12 weeks, included in participants’ regular diet in place of an equivalent food in terms of energy and macronutrient content. The nutritional contents of each HFC and LFC square (10 g) are presented in Additional file 1: Table S7. HFC provided 600 mg of flavanols daily.

HFC and LFC were supplied as chocolate bars by Barry-Callebaut, Lebeke-Wieze, Belgium. All steps of chocolate production (fermentation, drying, roasting, and alkalinization) were optimized to preserve antioxidants. Chocolate bars were standardized for their flavanol and theobromine content and matched for caloric load, nutrients and caffeine. They were similar in taste and colour and were supplied in individual, opaque packaging. 30 g of chocolate contained less than 25 mg of caffeine.

### Measurements

Recruited participants presented at the Institute of Nutrition and Functional Foods (INAF) clinical facility for a total of 10 visits, including 5 10-minute visits 24 hours after each main visit, for MED assessment.

Participants were asked to abstain from chocolate consumption, other than the study product, for the study’s duration, including the washout period, and for 7 days before the randomization visit. Intense physical activity was forbidden for 48 hours preceding each visit. Women could not apply any body lotion, gel or moisturizer on the skin for the 24 hours preceding each visit.

### Baseline

A short questionnaire documenting social and demographic characteristics, alcohol consumption, and medication, was completed by participants. Anthropometric data (body weight, height and body fat percentage) were measured according to a standard protocol. [26] Food habits and f1avonoid consumption during the last month were estimated by validated food frequency questionnaire (FFQ) [27]. Sun exposure (>30 minutes daily) and sun protection practices during the last summer and last week were evaluated by validated auto-administered questionnaire [28]. Blood samples were collected. MED and skin elasticity were measured, as were hydration parameters.

### Follow-up visits

Participants returned to our clinical research facility for follow-up visits at weeks 6, 9, and 12. All measurements at the 12^th^ week visit were repeated after a 3-week washout period (15^th^ week visit). MED, skin elasticity and hydration parameters were tested during each follow-up visit. Anthropometric data were collected. Blood samples were taken at every visit, in the morning after overnight fasting except for their morning 10-g intake of chocolate, for measurement of plasma flavanols and methylxanthines. Blood pressure was measured at every visit. Participants completed the FFQ to estimate their food consumption in the last month. Sun exposure and protection practices during the previous week were evaluated. Women returned 24 hours later for a 15-minute visit to assess the MED results.

### Evaluation of side-effects

BMI (kg/m^2^) and body fatness were assessed by bioelectrical impedance according to the validated Tanita technique. [26] Digestive and other symptoms (nausea, abdominal pain, constipation, and headache) were documented by questionnaire administered at randomization and at each study visit. Blood lipid profile and glucose were measured at baseline, at week 12 and after the 3-week washout period.

### Technical procedures

#### Minimal erythema dose

Defined as the lowest UV dose to elicit just perceptible erythema at 24 hours, MED was assessed by an automatic Durham erythema dose tester emitting narrowband ultraviolet B (UVB) light (emission peak 311 nm). This valid and reproducible method [29] involves a small hand-held unit containing low-pressure TL-01 tubes and a 10-aperture plate with metal foil attenuators designed to administer a 1.26 dose series (i.e. dose increments of 1.26 times the previous dose). The time period of MED tester application on the skin, corresponding to maximum dosage at open aperture, was determined by patients’ Fitzpatrick skin type. The attenuation factor of other apertures produced a dose sequence, with each subsequent hole receiving a smaller dose than the previous one. The test device was switched on for 10 minutes to reach optimum performance. After 10 minutes, the unit was switched off, and the test commenced immediately. Exposure time listed on previously-selected dosage was set and the device placed on a forearm (the same side was then used for all subsequent measures). The timer and tester were switched on simultaneously, and good skin contact was maintained. When the alarm sounded, the tester was switched off. All tests on each patient were performed at the same vertical level. MED was evaluated clinically under controlled, artificial lighting by determining which aperture presented just perceptible erythema 24 hours after irradiation, in accordance with a validated method for MED testing with the Durham erythema dose system [30-34].

### Skin elasticity parameters

Skin parameters were assessed by Cutometer (MPA580, Courage & Khazaka, Cologne, Germany) [35]. Measurements were based on the suction method. Negative pressure was created in the device, and skin was drawn into the probe’s aperture. Penetration depth was ascertained by a non-contact optical system consisting of a light source and light receiver as well as 2 prisms facing each other, projecting light from the transmitter to the receiver. Light intensity varied with penetration depth. With larger probe apertures, deeper layers of the skin are deformed by suction. We, therefore, chose an 8-mm aperture. The resistance of skin sucked up by negative pressure (firmness) and its ability to return to its original position (elasticity) were displayed as curves at the end of each measurement. The cutometer generated a graph (Additional file 1: Figure S1) depicting immediate deformation or skin extensibility (Ue), delayed distention (Uv), final deformation (Uf) and immediate retraction (Ur). Ur/Ue ratio, or net elasticity, was the parameter of choice for quantifying skin aging, since it represented the ability of skin to recover after deformation. This parameter is independent of skin thickness. We evaluated final skin distension (distensibility), overall elasticity and net e1asticity [36].

Before skin measurement, the participants remained in a seated position for 10 minutes, in an environmentally-controlled room (temperature: 22 ± 2°C, relative humidity: 40-60%), for acclimatization to ambient conditions.

### Skin hydration parameters

The CM 825 Corneometer (Courage & Khazaka, Cologne, Germany), a well-established and accurate system, estimated skin surface hydration level. It is principally based on capacitance measurement of dielectric media. Changes in the dielectric constant due to variation of skin surface hydration alter capacitance of the precision-measuring capacitor. Reproducibility of the instrument is high (coefficient of variation ± 3%). Measurement time is about 1 second. Even slight modifications of hydration level can be detected [37].

### Plasma flavanols and methylxanthines concentrations

Flavanols were measured in the INAF laboratory by P. Dubé. They were purified by solid extraction, followed by high-pressure liquid chromatography with a fluorescence detection system [38]. Methylxanthines were quantified by high-pressure liquid chromatography [39].

### Compliance

Study participants received a telephone reminder in the week preceding each visit. To promote chocolate compliance, the research coordinator telephoned them a few days before each study visit. A new appointment was scheduled if participants missed a visit. In addition, they documented their daily intake of chocolate bars on diary cards. Plasma theobromine concentrations served as marker of cocoa consumption [40].

### Blinding and methods for protecting against sources of bias

All clinical, biophysical, laboratory and statistical analyses were blinded. We applied the intent-to-treat principle to avoid attrition bias. Participants were compared in the groups to which they were originally assigned randomly. According to previous experience, we anticipated that 15% of randomized women would be lost to follow-up. To ensure support and motivation for substitution of equivalent foods by chocolate, participants received nutritional counselling at the randomization and 6^th^ week visits. All efforts were made to ensure primary outcome (MED) measurements at the 6^th^, 9^th^, 12^th^ and 15^th^ week visits in all randomized patients.

To avoid selection bias, the study subjects, investigators, staff and all laboratory analyses were blinded to treatment assignment. All chocolate bars were matched for calorie load, nutrients and caffeine. Similar in appearance (e.g. colour and quantity), smell and taste, they were supplied in individual, opaque packaging. The proportion of women who guessed right about group allocation was documented with a short questionnaire at the 15^th^ week visit.

To control for contamination bias, flavonoid consumption was measured by FFQ in the last month preceding each follow-up visit. Finally, data were collected according to a standardized procedure supervised by the research coordinator who had extensive experience in data monitoring. Sunbathing and tanning devices were not permitted during the study period.

### Sample size and planned recruitment rate

Williams et al. [12] reported MED mean ± standard error of the mean of 0.l09 ± 0.011 J/cm^2^ in a sample of healthy subjects, which rose to 0.223 ± 0.019 after 12-week chocolate intake. Based on these estimates, with standard deviation (SD) of 0.043, a sample of 31 women was required in each group to detect a minimal difference of 0.031 (28%) between groups with 5% 2-sided significance and 80% power. 15% loss to follow-up was anticipated so that sample size was increased to a total of 73 women. Augmenting it to 74 allowed equal numbers of subjects in both study arms.

### Statistical analysis

Statistical data analysis investigated the effect of HFC vs. LFC consumption on skin sensitivity to UV radiation according to MED criteria. Thus, the statistical hypothesis was that mean MED was not different in those who consumed HFC and those who did not: null hypothesis (H_0_): mean difference (MD) = 0. Statistical analysis was carried out at the St-François d'Assise Hospital Research Centre, CHUQ, with SAS software (version 9.3), according to the intent-to-treat principle. P values (bilateral) lower than or equal to 0.05 indicated significant differences. The baseline characteristics of participants in each group were compared by Chi-square test for categorical variables and independent sample t-test for continuous variables with normal distributions. These tests validated the randomization process. The primary outcome was changes in MED values, calculated as the difference for each person at weeks 6, 9 and 12 compared to baseline. MED at 15 weeks was compared to week 12. The secondary endpoints were assessed similarly. The primary outcome was analyzed in secondary analysis of repeated measures adjusted for potential factors affecting MED. Differences in the number and quality of side-effects between the 2 groups were compared as well.

The results were expressed as means ± SD at baseline and at 6, 9, 12 and 15 weeks; *n* was number of women. Missing data were imputed by a commonly-employed single imputation method with null values for mean differences. We explored different approaches to the imputation problem. All analyses are in general agreement qualitatively, and intent-to-treat results are presented. Multivariate analysis included baseline values for each outcome, Fitzpatrick skin phototype (I or II), season of study participation, age of participants (<50 years or ≥50 years), and BMI (for the skin hydration outcome only), as potential confounding variables. In the final multivariate model, baseline values for each analyzed outcome, season of study participation, age of participants (<50 years or ≥50 years), and BMI (for the skin hydration outcome only) were retained by backward step-wise elimination. If, by removing a variable from the model, change in the regression coefficient was more than 10% compared to the adjusted model including all variables, then that variable was retained in the final model. Skin phototype was not specifically accounted for in the final model for primary outcome since more precise measurement of skin photosensitivity was included in the form of MED values at baseline.

## Results

**Figure 1:**
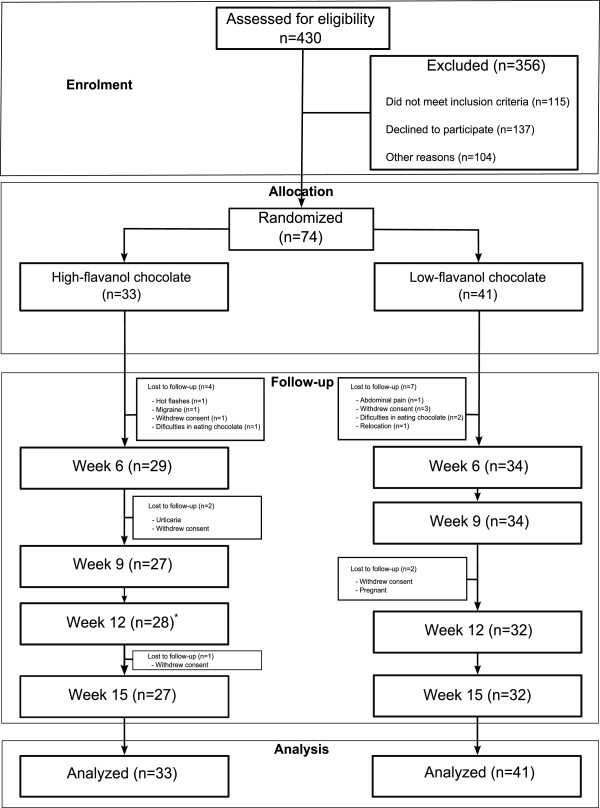
**Flow diagram of study participation.** *1 participant withdrew voluntarily from the study at week 9, but accepted to return at week 12.

For each group, Figure 1 shows the number of participants who were excluded before randomization, or were randomly assigned, received intended treatment or were lost after randomization with reasons, and were analyzed for primary and secondary outcomes.

Of the 74 randomized study participants, 59 (27 and 32 in the HFC and LFC arms, respectively) had outcomes available for analysis per protocol. Intent-to-treat analysis was conducted as described above. 7 and 9 women respectively were lost to follow-up for reasons not related to the intervention.

At randomization, the 2 arms were well balanced with regard to social and baseline characteristics, excluding BMI which was 1.8 higher in the LFC group and season at week 12. Specifically, the percentage of participants in the HFC group who were evaluated before the spring season was lower than in the LFC group (Table 1).

**Table 1:** Baseline and demographic characteristics

**Clinical characteristics**	**High-flavanol chocolate (n = 33)**	**Low-flavanol chocolate (n = 41)**
**MED (J/cm**^**2**^**)**	0.56 ± 0.14	0.61 ± 0.17
**Skin type**^**˦ **^**I**	15 (45.5)	16 (39.0)
**II**	18 (54.5)	25 (61.0)
**Season at week 12**^**˦**^		
Before April 15	10 (30.3)	17 (41.5)
After April 15	23 (69.7)	24 (58.5)
**Overall skin elasticity (mm)**		
Arm	0.76 ± 0.08	0.76 ± 0.09
Temple	0.54 ± 0.11	0.55 ± 0.10
Skin hydration (units^**^)		
Arm	33.34 ± 7.27	33.05 ± 6.76
Temple	41.00 ± 13.02	42.39 ± 10.71
**Plasma polyphenol concentrations**		
Epicatechins (ng/ml)	0.12 ± 0.48	0.11 ± 0.43
Catechins (ng/ml)	0.20 ± 0.67	0.17 ± 0.64
Dietary polyphenol consumption*		
Epicatechins (mg/day)	10.50 ± 7.54	10.65 ± 7.00
Catechins (mg/day)	11.94 ± 7.91	14.35 ± 9.28
**Age (years)**	39.7 ± 14.2	39.3 ± 12.0
20 to 35^**˦**^	15 (45.5)	19 (46.3)
36 to 49^**˦**^	8 (24.2)	12 (29.3)
50 to 65^**˦**^	10 (30.3)	10 (24.4)
**Sun exposure habits**^**˦**^		
UV exposure (>30 minutes daily):		
Weekdays	23 (69.7)	30 (73.2)
Weekend days	31 (93.9)	39 (95.1)
Painful sunburn	23 (69.7)	28 (68.3)
**Educational attainment**^**˦**^		
University degree	24 (72.7)	36 (87.8)
Some university or college education	9 (27.3)	5 (12.2)
**Anthropometric measures**		
BMI (kg/m^2^)	22.6 ± 2.2	24.4 ± 3.6

**Arbitrary Corneometer® units. Values represent means ± SD.

*Polyphenol consumption was calculated by FFQ.

^**˦**^Values represent number and percentage.

### Primary outcome

#### Minimal erythema dose

**Figure 2:**
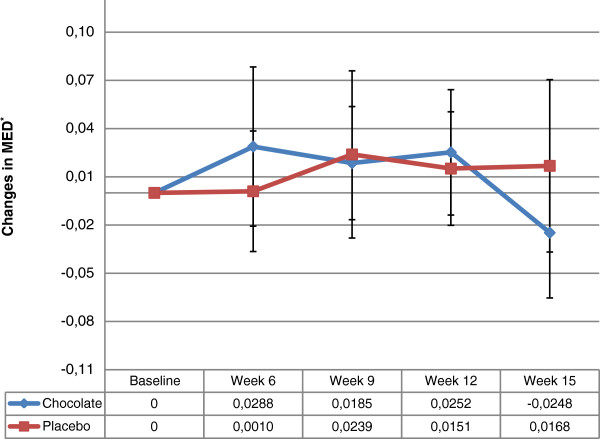
**Mean MED differences within treatments.** *Changes in MED corresponded to differences between the endpoints evaluated (6, 9, 12 and 15 weeks) and baseline (J/cm^2^).

Table 2 reports that HFC consumption had no effect on MED at 6, 9 and 12 weeks in comparison to control LFC. After the 3-week washout period, MED in the HFC group receded to baseline. On the other hand, MED in the LFC arm continued to increase. Figure 2 illustrates the tendency of mean MED differences after daily consumption of 30 g chocolate. The nearly parallel curves for both groups up to week 12 illustrated the absence of a significant difference in MED at this time-point. However, both groups went in opposite directions, starting from the washout period.

**Table 2:** MED after daily consumption of 30 g chocolate for 6, 9 and 12 weeks and after 3-week washout

	**High-flavanol chocolate (n = 33)**	**Low-flavanol chocolate (n = 41)**	***P *** **value of changes between treatments**
MED (J/cm^2^)			
Baseline	0.56 ± 0.14	0.61 ± 0.17	-
Week 6	0.59 ± 0.17	0.61 ± 0.15	0.36
Week 9	0.58 ± 0.15	0.63 ± 0.18	0.86
Week 12	0.58 ± 0.14	0.62 ± 0.18	0.70
*P* value (Week 6 *vs.* Baseline)	0.24	0.96	
*P* value (Week 9 *vs.* Baseline)	0.29	0.36	
*P* value (Week 12 *vs.* Baseline)	0.20	0.39	
MED Washout period			
Week 15	0.56 ± 0.14	0.64 ± 0.18	0.21
*P* value (Week 15 *vs.*Week 12)	0.22	0.53	

Values represent means ± SD.

Multivariate analysis for adjusted changes in MED at 6, 9 and 12 weeks gave findings similar to those with univariate analysis. Conversely, the difference between adjusted changes in MED after the washout period in the HFC arm was -0.07 higher and in the opposite direction compared to the LFC group (MD = -0.07; 95% confidence interval (CI): -0.13 to -0.01; *P* = 0.01; R^2^ = 0.46) (Additional file 1: Table S1), thus becoming statistically significant. Age ≥50 years was associated with greater changes in MED at 12 and 15 weeks, and spring season of evaluation was linked with lesser changes in MED at 12 and 15 weeks (Additional file 1: Table S2).

### Secondary endpoints

#### Skin hydration and elasticity parameters

Overall temple elasticity increased slightly but significantly: 0.05 ± 0.09 mm in the HFC group at 6 weeks compared to LFC (*P* = 0.04) (Table 3). Net temple elasticity also increased slightly but significantly: 0.08 ± 0.08 and 0.09 ± 0.12 mm in the HFC group at 6 and 12 weeks, respectively, compared to LFC (*P* = 0.01 and *P* = 0.03, respectively) (Table 4). No other significant between-group changes were observed (Tables 3 and 4 and Additional file 1: Tables S8 and S9).

**Table 3:** Overall skin elasticity after daily consumption of 30 g chocolate for 6, 9 and 12 weeks

	**High-flavanol chocolate (n = 33)**	**Low-flavanol chocolate (n = 41)**	***P *** **value of changes between treatments**
Overall elasticity (mm)			
Arm			
Baseline	0.76 ± 0.08	0.76 ± 0.09	-
Week 6	0.76 ± 0.09	0.75 ± 0.06	0.61
Week 9	0.77 ± 0.08	0.76 ± 0.07	0.48
Week 12	0.76 ± 0.10	0.75 ± 0.08	0.42
*P* value (Week 6 *vs* Baseline)	0.97	0.51	
*P* value (Week 9 *vs* Baseline)	0.29	0.95	
*P* value (Week 12 *vs* Baseline)	0.77	0.39	
Temple			
Baseline	0.54 ± 0.11	0.55 ± 0.10	-
Week 6	**0.59 ± 0.09**	**0.55 ± 0.10**	**0.04**
Week 9	0.55 ± 0.12	0.56 ± 0.10	0.64
Week 12	0.60 ± 0.12	0.57 ± 0.11	0.10
*P* value (Week 6 *vs* Baseline)	0.0024	0.92	
*P* value (Week 9 *vs* Baseline)	0.33	0.81	
*P* value (Week 12 *vs* Baseline)	0.0016	0.10	

Values represent means ± SD.

Values in bold are statistically significant.

**Table 4:** Net skin elasticity after daily consumption of 30 g chocolate for 6, 9 and 12 weeks

	**High-flavanol chocolate (n = 33)**	**Low-flavanol chocolate (n = 41)**	***P *** **value of changes between treatments**
Net elasticity (mm)			
Arm			
Baseline	0.73 ± 0.19	0.75 ± 0.20	-
Week 6	0.75 ± 0.18	0.78 ± 0.20	0.52
Week 9	0.77 ± 0.21	0.81 ± 0.22	0.49
Week 12	0.81 ± 0.22	0.80 ± 0.23	0.54
*P* value (Week 6 *vs* Baseline)	0.50	0.12	
*P* value (Week 9 *vs* Baseline)	0.07	0.01	
*P* value (Week 12 *vs* Bbaseline)	0.02	0.13	
			
Temple			
Baseline	0.43 ± 0.14	0.45 ± 0.13	-
Week 6	**0.51 ± 0.15**	**0.47 ± 0.13**	**0.01**
Week 9	0.48 ± 0.17	0.47 ± 0.13	0.41
Week 12	**0.52 ± 0.19**	**0.48 ± 0.12**	**0.03**
*P* value (Week 6 *vs* Baseline)	<.0001	0.12	
*P* value (Week 9 *vs* baseline)	0.02	0.38	
*P* value (Week 12 *vs* Baseline)	0.0002	0.21	

Values represent means ± SD.

Values in bold are statistically significant.

Multivariate analysis of adjusted changes in skin hydration and elasticity parameters at 6, 9 and 12 weeks, and after 3-week washout gave results comparable to those of univariate analysis (Additional file 1: Tables S3-S6).

### Plasma polyphenol concentrations

Plasma polyphenol concentrations were similar in both groups at baseline. The LFC group presented clinically small but statistically significant increases in plasma epicatechins at weeks 6, 9 and 12 compared to baseline (*P* < 0.0001). In the HFC group, marked increments in plasma epicatechin concentrations were clinically and statistically significant (*P* < 0.0001) compared to baseline at weeks 6, 9, and 12. However, plasma concentration at week 12 was lower than at week 6. Changes in plasma epicatechin concentrations in the HFC group were statistically higher than in the LFC group at all measurement times after baseline (*P* < 0.0001). Changes in plasma catechins tended to be similar (Additional file 1: Table S10).

### Plasma methylxanthine concentrations

Plasma theobromine, theophylline and caffeine concentrations increased in both groups at weeks 6, 9 and 12 versus baseline. However, no difference was found in changes of plasma methylxanthine concentrations between the HFC and LFC groups at all measurement times (Additional file 1: Table S11).

### Consumption of dietary polyphenols

Consumption of dietary polyphenols was similar between both groups at baseline. During the study period, both groups tended towards decreased dietary polyphenols (not including interventional chocolate consumption). No differences were evident in changes of dietary polyphenol consumption between the HFC and the LFC groups (Additional file 1: Table S12).

### Washout period

Skin distensibility decreased slightly but significantly (-0.02 ± 0.03 mm) in the HFC group at 15 weeks after washout compared to LFC (*P* = 0.04) (Additional file 1: Table S13). Plasma polyphenol concentrations returned to baseline after the washout period in both groups. This change was statistically different when both groups were compared (*P* < 0.0001 and *P* = 0.0005 for epicatechins and catechins, respectively) (Additional file 1: Table S14). Plasma methylxanthine concentrations decreased to baseline after 3-week washout. The change was not statistically different between the 2 groups (*P* = 0.07, *P* = 0.69 and *P* = 0.10 for theobromine, theophylline and caffeine, respectively) (Additional file 1: Table S15). No other significant between-group differences were observed in skin hydration and dietary polyphenol consumption during the washout period (Additional file 1: Tables S16 and S17).

## Discussion

As UV radiation accelerates skin aging and promotes skin cancer, novel photoprotective measures represent a promising area of research. Dietary polyphenols are natural antioxidant and anti-inflammatory agents that could protect skin against UV exposure. In our population of healthy non-smoking women, 12-week HFC intake was associated with significantly increased net skin elasticity but no significant change in MED. Participants did not report any significant clinical side-effects.

The current literature suggests that chronic ingestion of HFC may be photoprotective, but there are several methodological limitations. Only 1 controlled, double-blind, RCT evaluated the effect of HFC vs. LFC intake on skin sensitivity to UV radiation after 12 weeks, as measured by MED [23]. 22 women and 8 men with Fitzpatrick skin phototypes II or III were included in that trial. After 12 weeks, mean MED remained stable in the LFC group compared to baseline. Statistically significant increases in mean MED were observed with HFC (within-group comparisons). Nevertheless, these authors did not report between-group comparisons. Moreover, women and men were included in their trial, but participants’ characteristics in each group were not elaborated. Finally, our study population was quite different from that of Williams’ [23] as we included only women with skin type I or II.

Heinrich et al. enrolled 24 women in their RCT and evaluated the effect of HFC vs. LFC intake on MED in solar simulation radiation [21]. After 12 weeks, women assigned to the HFC group showed significantly increased MED in comparison to the LFC group. It is important to note that the characteristics of women in each group were not presented, and no information was given regarding trial profile (loss-to-follow-up, intent-to-treat analysis). Finally, visually-assessed MED, considered to be the gold standard [41], was not available. Thus, direct comparison with other studies was not possible.

Several factors could have contributed to our finding of no statistically photoprotective effects of HFC vs. LFC measured by MED at 12 weeks. Season of participation differed in the 2 groups. Specifically, more participants in the LFC group started the trial during winter and finished at the 12-week endpoint during the spring season, potentially introducing a residual confounding effect. During this period, physiologically-increased MED is attributed to longer solar exposure [42,43]. Indeed, exploratory analyses of our data demonstrated a boost in MED during spring, compared to its stability during winter and, inversely, a decrease during fall (Additional file 1: Figure S2). This extrinsic effect could partly explain the shape of the LFC group curve in Figure 2, in which it can be seen that MED increased from baseline to week 12. Consequently, the increment could have masked difference from the HFC group. Differing season of participation could also have introduced bias in the results, since roughly one-third of participants in each group completed the study during spring, when the natural increase in MED could have rendered the photoprotective effects of chocolate less visible. Indeed, multivariate analyses revealed that HFC group participants terminating during spring manifested lower changes in MED. Furthermore, it would have been interesting to study women over the age of 50 years, as our multivariate analyses disclosed greater changes in MED in this population, indicating that HFC may have significant photoprotective effects after menopause in comparison to control LFC. Finally, MED imbalance at baseline rendered our groups less comparable, which we might not have completely accounted for despite adjustments in our multivariate model.

Lack of compliance with chocolate consumption is another factor that could partially explain our non-significant results. In fact, the HFC group showed a stronger increase in MED at week 6 than at weeks 9 and 12 compared to baseline, mimicking the same tendency found in plasma polyphenol concentrations at these time periods. Women could have been slightly less compliant with chocolate consumption after 6 weeks, rendering its effects even less noticeable.

It would have been interesting to compare HFC with LFC low in theobromine, the primary alkaloid in cocoa. Theobromine concentrations in our LFC and HFC groups were similar. Although it has never been studied extensively in humans, sparse data indicate that it has been tested for the treatment of hypertension to exploit its vasodilator and smooth tissue-relaxing properties [44,45]. Theobromine may contribute to the effect of dark chocolate on endothelium function. Therefore, it could have increased the microcirculatory delivery of flavanols in both groups (as the LFC group also showed significant flavanol elevation), and masked the isolated action of polyphenols.

Moreover, since MED was highly variable between women participating in our clinical trial (as relative SD was 25%), the non-optimal power of our study sample could partially explain the non-significant results. Furthermore, the variability of our results is highlighted by the large CIs revealed by multivariate analysis. In addition, it’s not possible to exclude that imbalance in numbers allocated to HFC and LFC partly explained by the 2 independently generated randomisation lists and differences between the 2 groups for skin type and date of entry could somewhat bias our results. Indeed, as specified in the section methods, after three months of recruitment, proportion of women with skin type 2 and age 50–65 were more prevalent than expected and a new independent list of randomisation was generated. Moreover, in the adjusted model, age group and skin type didn’t modify the results.Interestingly, after 3-week washout, the HFC group showed decreased MED with return to baseline. Conversely, the LFC group manifested continuously elevated MED. We would, therefore, have expected similar increment in the HFC group, instead of the observed decline. This differential tendency after removal of our intervention, well-illustrated in Figure 2, indicates loss of a clinical effect too small to be statistically significant due to the afore-mentioned factors contributing to lack of significance.

MED represents the lowest UV dose necessary to produce just perceptible erythema at 24 hours. The erythema reaction to UV is the endpoint of complex biological processes, including direct DNA, lipid and protein damage, activation of pro-inflammatory pathways and generation of free radicals, mainly reactive oxygen species. Polyphenols are expected to act mainly on the latter by supplementing the body’s natural, free-radical-quenching, antioxidant mechanisms [10-14] . A recently-published review [46] of photoprotection and antioxidants notes that the action spectrum of reactive oxygen species generation is predominately in the ultraviolet A (UVA) range, although there is some overlap with UVB. Thus, perhaps antioxidants play a larger role in protecting against UVA-induced production of free radicals. Consequently, measurement of the photoprotective effects of flavanols solely by MED after exposure to UVB might not be fully representative of their real biological potential in this regard [46].

A positive outcome of HFC consumption was noted in net temple elasticity at 12 weeks compared to LFC, but was not reproduced in arm elasticity, suggesting that flavanols might impact only sun-exposed skin for this outcome. Furthermore, the gain in elasticity was not lost after 3-week washout. The underlying mechanisms are not known, but augmented blood flow [24] can lead to heightened production of collagen and elastin, key structural proteins which diminish in the skin with aging. On the other hand, HFC consumption did not modify skin hydration. Heinrich et al. postulated improvements in skin density and thickness, stratum corneum hydration, transepidermal water loss, and skin surface roughness after 12 weeks of flavanol-rich cocoa drink consumption in healthy women [21].

HFC and LFC consumption did not significantly change BMI and blood glucose, a previously-reported finding [47]. Lipid profile also was not affected, except for a slight but significant rise in high-density lipoprotein-cholesterol level in the HFC compared to the LFC group at week 12. Participants did not report any significant clinical side-effects. In all, chocolate consumption did not result in notable adverse events.

## Conclusion

We believe that additional clinical trials are needed to fully evaluate the photoprotection conferred by HFC consumption. Exploration of this association by our study highlights the importance of sufficient sample size in the context of great inter-individual variability in skin sensitivity to UV, and perhaps the use of solar simulators to more accurately assess the protective effects of flavanols. In addition, all participants should be randomized according to MED to render groups comparable, and evaluated in a single season. Flavanol effects could also be explored in women after menopause. Finally, the effect of HFC consumption compared with no (or usual/discretionary) chocolate consumption on MED remains to be addressed.

## Abbreviations

BMI: Body mass index; CB: Catherine Boivin; CI: Confidence interval; CLB: Claudine Blanchet; FFQ: Food frequency questionnaire; H_0_: Null hypothesis; HFC: High-flavanol chocolate; HRT: Hormone replacement therapy; INAF: Institute of Nutrition and Functional Foods; JAM: Jaime Andres Mogollon; JC: Joël Claveau; LFC: Low-flavanol chocolate; DM: Mean difference; MED: Minimal erythema dose; RCT: Randomized controlled trial; SD: Standard deviation; SL: Simone Lemieux; SYD: Sylvie Dodin; Ue: Immediate deformation or skin extensibility; Uf: Final deformation; Ur: Immediate retraction; Uv: Delayed distention; UV: Ultraviolet; UVA: Ultraviolet A; UVB: Ultraviolet B.

## Competing interests

All authors declare that they have no conflicts of interest.

## Authors’ contributions

JAM and CB performed the experiments, collected, analyzed and interpreted the data, and wrote the manuscript. CLB conceived, designed and coordinated the experiments and critically revised the text. SL and JC collected data and provided critical revision. SYD conceived and designed the study, analyzed and interpreted the data, supervised writing of the paper and made critical revisions. All authors read and approved the final manuscript.

## Authors’ information

JAM is a nutritionist and PhD candidate in nutrition at Université Laval. CB is a senior resident in dermatology at Université Laval. SL is professor in food science and nutrition at Université Laval and researcher at the Institute of Nutrition and Functional Foods (INAF). CLB is a PhD research assistant at Université Laval with special skills in systematic reviews. JC is a dermatologist and professor in melanoma and skin cancer at Université Laval and clinician-scientist at the Melanoma and Skin Cancer Clinic, Dermatology Division, Hôtel-Dieu Hospital, CHUQ, Quebec. SYD is professor in obstetrics and gynecology at Université Laval and clinician-scientist at CHUQ, with expertise in randomized clinical trials.

## Supplementary Material

Additional file 1 Chocolate flavanols and skin photoprotection: a parallel, double-blind, randomized clinical trial. Click here for file
