# Supplementary material for: Chocolate flavanols and skin photoprotection: a parallel, double-blind, randomized clinical trial
Source: Nutr J. 2014 Jun 27;13:66. doi: 10.1186/1475-2891-13-66 (PMC4082621; doi:10.1186/1475-2891-13-66)

Table S1. Adjusted changes in MED after daily consumption of 30 g chocolate for 6, 9 and 12 weeks and after 3-week washout

	Non-adjusted			Adjusted*		
	$^{\Delta}$ Change in MED	*95% CI*	*P* value**	$^{\beta}$ Change in MED	*95% CI*	*P* value
Changes in MED (J/cm^2)						
Baseline***	-0.051 ± 0.036	-0.124 - 0.021	0.16	-0.058$^{£}$ ± 0.034	-0.127 - 0.010	0.09
Week 6	0.028 ± 0.030	-0.032 - 0.088	0.36	0.008 ± 0.029	-0.049 - 0.066	0.77
Week 9	-0.005 ± 0.033	-0.070 - 0.060	0.88	-0.029 ± 0.031	-0.091 - 0.033	0.35
Week 12	0.010 ± 0.026	-0.042 - 0.062	0.70	-0.004 ± 0.027	-0.058 - 0.049	0.87
Changes in MED Washout period						
Week 15	-0.042 ± 0.035	-0.111 - 0.027	0.23	**-0.077$^{££}$ ± 0.030**	**-0.136 - -0.017**	**0.01**

*Values were adjusted for MED at baseline, age of participants (<50 years or ≥50 years) and season of study participation (before or after April 15)

$^{\Delta}$: Differences in absolute MED changes between treatments

$^{\beta}$: Linear regression coefficient of absolute change in MED

**Between treatments

***Differences between treatments in baseline MED values

$^{£}$ Values were adjusted for age of participants (<50 years or ≥50 years)

$^{££}$ Values were adjusted for MED at week 12, age of participants (<50 years or ≥50 years) and season of study participation (before or after April 15)

Values represent means ± standard error (SE)

Table S2. Multivariate analyses comparing changes in MED between high-flavanol and low-flavanol chocolate arms after 3-week washout

	Non-adjusted		
	[β] Change in MED	*95% CI*	*P* value
Intercept	0.368 ± 0.066	0.237 - 0.498	<0.001
Intervention	-0.077 ± 0.030	-0.136 - 0.017	0.01
MED week 12	0.456 ± 0.097	0.263 - 0.649	<0.001
Age (≥50 versus <50 years)	0.082 ± 0.036	0.011 - 0.153	0.02
Season of study participation (before versus after April 15)	-0.072 ± 0.031	-0.135 - -0.010	0.02
R^2	0.46		

*Values were adjusted for MED at baseline, age of participants (<50 years or ≥50 years) and season of study participation (before or after April 15)

[β]: Linear regression coefficient of absolute change in MED

Values represent means ± SE

Table S3. Adjusted changes in skin distensibility after daily consumption of 30 g chocolate for 6, 9 and 12 weeks and after 3-week washout

	Non-adjusted			Adjusted*		
	Δ Change in MED	*95% CI*	*P* value**	β Change in MED	*95% CI*	*P* value
Skin distensibility (mm)						
Arm						
Baseline***	0.009 ± 0.018	-0.026 - 0.044	0.60	0.013£ ± 0.017	-0.021 - 0.048	0.45
Week 6	0.004 ± 0.008	-0.013 - 0.021	0.65	0.006 ± 0.008	-0.009 - 0.021	0.45
Week 9	0.006 ± 0.008	-0.010 - 0.022	0.44	0.007 ± 0.008	-0.009 - 0.022	0.39
Week 12	0.009 ± 0.010	-0.010 - 0.029	0.33	0.011 ± 0.009	-0.006 - 0.028	0.22
Changes in skin distensibility (mm) Washout period						
Week 15	-0.012 ± 0.006	-0.024 - -0.001	0.04	-0.010££ ± 0.006	-0.022 - 0.002	0.10
Temple						
Baseline***	0.007 ± 0.014	-0.021 - 0.035	0.62	0.011£ ± 0.014	-0.016 - 0.038	0.43
Week 6	-0.001 ± 0.013	-0.024 - 0.022	0.92	0.002 ± 0.011	-0.019 - 0.024	0.83
Week 9	-0.005 ± 0.012	-0.029 - 0.019	0.70	-0.004 ± 0.011	-0.027 - 0.019	0.72
Week 12	-0.003 ± 0.010	-0.023 - 0.018	0.79	0.000 ± 0.010	-0.019 - 0.020	0.98
Changes in skin distensibility (mm) Washout period						
Week 15	-0.001 ± 0.010	-0.020 - 0.018	0.89	-0.001££ ± 0.010	-0.020 - 0.018	0.91

*Values were adjusted for skin distensibility at baseline, age of participants (<50 years or ≥50 years), Fitzpatrick skin phototype (I or II) and season of study participation (before or after April 15)
Δ: Differences in absolute changes in skin distensibility between treatments
β: Linear regression coefficient of absolute change in skin distensibility
**Between treatments
***Differences in skin distensibility at baseline between treatments
£ Values were adjusted for age of participants (<50 years or ≥50 years) and Fitzpatrick skin phototype (I or II)
££ Values were adjusted for skin distensibility at week 12, age of participants (<50 years or ≥50 years), Fitzpatrick skin phototype (I or II) and season of study participation (before or after April 15)
Values represent means ± SE

Table S4. Adjusted changes in overall elasticity after daily consumption of 30 g chocolate for 6, 9 and 12 weeks and after 3-week washout

	Non-adjusted			Adjusted*		
	[Δ] Change in MED	*95% CI*	*P* value**	[β] Change in MED	*95% CI*	*P* value
Overall elasticity (mm)						
Arm						
Baseline***	-0.002 ± 0.021	-0.043 - 0.040	0.94	0.003[£] ± 0.020	-0.037 - 0.044	0.87
Week 6	0.010 ± 0.019	-0.029 - 0.048	0.62	0.010 ± 0.016	-0.021 - 0.042	0.51
Week 9	0.011 ± 0.015	-0.019 - 0.040	0.48	0.013 ± 0.013	-0.013 - 0.038	0.32
Week 12	0.017 ± 0.021	-0.024 - 0.058	0.42	0.016 ± 0.018	-0.019 - 0.052	0.36
Changes in overall elasticity (mm)	Washout period					
Week 15	-0.010 ± 0.015	-0.040 - 0.019	0.50	-0.001[££] ± 0.013	-0.026 - 0.027	0.97
Temple						
Baseline***	-0.013 ± 0.025	-0.063 - 0.037	0.60	-0.014[£] ± 0.025	-0.065 - 0.036	0.57
Week 6	0.052 ± 0.026	0.000 - 0.104	0.05	**0.046 ± 0.021**	**0.001 - 0.088**	**0.03**
Week 9	0.012 ± 0.026	-0.039 - 0.063	0.64	0.010 ± 0.022	-0.035 - 0.055	0.65
Week 12	0.035 ± 0.021	-0.007 - 0.077	0.10	0.045 ± 0.018	0.008 - 0.081	0.02
Changes in overall elasticity (mm)	Washout period					
Week 15	-0.018 ± 0.020	-0.058 - 0.022	0.37	-0.001[££] ± 0.018	-0.037 - 0.035	0.95

*Values were adjusted for skin distensibility at baseline, age of participants (<50 years or ≥50 years), Fitzpatrick skin phototype (I or II) and season of study participation (before or after April 15)

[Δ]: Differences in absolute changes in overall elasticity between treatments

[β]: Linear regression coefficient of absolute change in overall elasticity

**Between treatments

***Differences in overall elasticity at baseline between treatments

[£] Values were adjusted for age of participants (<50 years or ≥50 years) and Fitzpatrick skin phototype (I or II)

[££] Values were adjusted for overall elasticity at week 12, age of participants (<50 years or ≥50 years), Fitzpatrick skin phototype (I or II) and season of study participation (before or after April 15)

Values represent means ± SE

Table S5. Adjusted changes in net elasticity after daily consumption of 30 g chocolate for 6, 9 and 12 weeks and after 3-week washout

	Non-adjusted			Adjusted*		
	$^{\Delta}$ Change in MED	*95% CI*	*P* value**	$^{\beta}$ Change in MED	*95% CI*	*P* value
Net elasticity (mm)						
Arm						
Baseline***	-0.017 ± 0.046	-0.109 - 0.075	0.71	-0.011$^{£}$ ± 0.046	-0.102 - 0.081	0.82
Week 6	-0.019 ± 0.029	-0.077 - 0.040	0.52	-0.021 ± 0.028	-0.076 - 0.035	0.46
Week 9	-0.023 ± 0.033	-0.088 - 0.043	0.49	-0.016 ± 0.031	-0.079 - 0.046	0.60
Week 12	0.028 ± 0.046	-0.063 - 0.119	0.54	0.033 ± 0.044	-0.054 - 0.120	0.46
Changes in net elasticity (mm) Washout period						
Week 15	-0.004 ± 0.038	-0.080 - 0.071	0.90	0.019$^{££}$ ± 0.036	-0.052 - 0.090	0.60
Temple						
Baseline***	-0.019 ± 0.031	-0.080 - 0.043	0.55	-0.023$^{£}$ ± 0.031	-0.084 - 0.039	0.46
Week 6	0.050 ± 0.020	0.010 - 0.090	0.01	**0.050 ± 0.020**	**0.011 - 0.090**	**0.01**
Week 9	0.023 ± 0.029	-0.033 - 0.078	0.41	0.023 ± 0.027	-0.031 - 0.078	0.39
Week 12	0.065 ± 0.028	0.008 - 0.121	0.03	**0.072 ± 0.027**	**0.019 - 0.126**	**0.01**
Changes in net elasticity (mm) Washout period						
Week 15	-0.048 ± 0.024	-0.096 - -0.001	0.05	-0.035$^{££}$ ± 0.024	-0.083 - 0.013	0.15

*Values were adjusted for skin distensibility at baseline, age of participants (<50 years or ≥50 years), Fitzpatrick skin phototype (I or II) and season of study participation (before or after April 15)

$^{\Delta}$: Differences in absolute changes in net elasticity between treatments

$^{\beta}$: Linear regression coefficient of absolute change in net elasticity

**Between treatments

***Differences in net elasticity at baseline between treatments

$^{£}$ Values were adjusted for age of participants (<50 years or ≥50 years) and Fitzpatrick skin phototype (I or II)

$^{££}$ Values were adjusted for net elasticity at week 12, age of participants (<50 years or ≥50 years), Fitzpatrick skin phototype (I or II) and season of study participation (before or after April 15)

Values represent means ± SE

Table S6. Adjusted changes in skin hydration after daily consumption of 30 g chocolate for 6, 9 and 12 weeks and after 3-week washout

	Non-adjusted			Adjusted*		
	Δ Change in MED	*95% CI*	*P* value**	β Change in MED	*95% CI*	*P* value
Skin hydration						
Arm						
Baseline***	0.293 ± 1.634	-2.965 - 3.552	0.86	0.863 ± 1.711	-2.550 - 4.276	0.62
Week 6	1.032 ± 1.425	-1.808 - 3.874	0.47	1.032 ± 1.425	-1.808 - 3.874	0.47
Week 9	-2.571 ± 1.487	-5.536 - 0.393	0.09	-1.160 ± 1.290	-3.734 - 1.414	0.37
Week 12	-1.757 ± 1.815	-5.374 - 1.861	0.34	-0.720 ± 1.767	-4.247 - 2.807	0.68
Changes in skin hydration Washout period						
Week 15	0.946 ± 1.510	-2.064 - 3.956	0.53	0.160 ± 1.739	-2.593 - 2.913	0.91
Temple						
Baseline***	-1.394 ± 2.758	-6.892 - 4.103	0.61	-0.688 ± 2.794	-6.262 - 4.886	0.81
Week 6	-1.561 ± 1.847	-5.243 - 2.121	0.40	-2.603 ± 1.746	-6.088 - 0.882	0.14
Week 9	1.081 ± 2.046	-2.998 - 5.160	0.60	1.473 ± 1.961	-2.441 - 5.387	0.45
Week 12	1.732 ± 2.131	-2.517 - 5.980	0.42	1.684 ± 1.872	-2.052 - 5.421	0.37
Changes in skin hydration Washout period						
Week 15	-0.461 ± 1.900	-4.249 - 3.327	0.81	-0.137 ± 1.909	-3.948 - 3.673	0.94

*Values were adjusted for skin hydration at baseline, age of participants (<50 years or ≥50 years), Fitzpatrick skin phototype (I or II) and season of study participation (before or after April 15) and BMI at baseline

Δ: Differences in absolute changes in skin hydration between treatments

β: Linear regression coefficient of absolute change in skin hydration

**Between treatments

***Differences in skin hydration at baseline between treatments

£ Values were adjusted for age of participants (<50 years or ≥50 years) and Fitzpatrick skin phototype (I or II)

££ Values were adjusted for skin hydration at week 12, age of participants (<50 years or ≥50 years), Fitzpatrick skin phototype (I or II) and season of study participation (before or after April 15) and BMI at week 12

Values represent means ± SE

Table S7. Chocolate composition (10 g)

Components	High-flavanol chocolate	Low-flavanol chocolate
Energy (kcal)	51	51
Total fat (g)	3.67	3.67
Carbohydrates (g)	3.72	3.72
Protein (g)	0.67	0.67
Total flavanols (mg)	200	< 30
Caffeine (mg)	7.5	7.5
Theobromine (mg)	75	75

Table S8. Skin distensibility after daily consumption of 30 g chocolate for 6, 9 and 12 weeks

	High-flavanol chocolate (n=33)	Low-flavanol chocolate (n=41)	*P* value of changes between treatments
Skin distensibility (mm)			
Arm			
Baseline	0.25 ± 0.07	0.24 ± 0.08	-
Week 6	0.22 ± 0.06	0.20 ± 0.07	0.65
Week 9	0.22 ± 0.07	0.20 ± 0.07	0.44
Week 12	0.21 ± 0.06	0.19 ± 0.07	0.34
P value (Week 6 *vs* Baseline)	0.0003	<.0001	
P value (Week 9 *vs* Baseline)	0.0002	<.0001	
P value (Week 12 *vs* Baseline)	<.0001	<.0001	
Temple			
Baseline	0.22 ± 0.06	0.22 ± 0.06	-
Week 6	0.21 ± 0.06	0.21 ± 0.06	0.92
Week 9	0.21 ± 0.07	0.21 ± 0.06	0.70
Week 12	0.21 ± 0.06	0.20 ± 0.06	0.79
P value (Week 6 *vs* Baseline)	0.25	0.46	
P value (Week 9 *vs* Baseline)	0.13	0.35	
P value (Week 12 *vs* Baseline)	0.03	0.03	

Values represent means ± SD

Table S9. Skin hydration after daily consumption of 30 g chocolate for 6, 9 and 12 weeks

	High-flavanol chocolate (n=33)	Low-flavanol chocolate (n=41)	*P* value of changes between treatments
Skin hydration (units**)			
Arm			
Baseline	33.34 ± 7.27	33.05 ± 6.76	-
Week 6	33.92 ± 7.41	32.59 ± 6.27	0.47
Week 9	31.32 ± 6.72	33.60 ± 6.55	0.09
Week 12	31.04 ± 8.54	32.51 ± 7.54	0.34
P value (Week 6 *vs* Baseline)	0.63	0.60	
P value (Week 9 *vs* Baseline)	0.04	0.62	
P value (Week 12 *vs* Baseline)	0.14	0.61	
Temple			
Baseline	41.00 ± 13.02	42.39 ± 10.71	-
Week 6	40.23 ± 11.30	43.18 ± 10.37	0.40
Week 9	40.60 ± 11.82	40.91 ± 10.72	0.60
Week 12	39.61 ± 10.31	39.27 ± 9.94	0.42
P value (Week 6 *vs* Baseline)	0.56	0.54	
P value (Week 9 *vs* Baseline)	0.77	0.32	
P value (Week 12 *vs* Baseline)	0.44	0.02	

** Arbitrary Corneometer® units
Values represent means ± SD

Table S10. Plasma polyphenol concentrations after daily consumption of 30 g chocolate for 6, 9 and 12 weeks

	High-flavanol chocolate (n=33)	Low-flavanol chocolate (n=41)	*P* value of changes between treatments
Epicatechins (ng/ml)			
Baseline	0.12 ± 0.48	0.11 ± 0.43	-
Week 6	78.28 ± 64.35	7.32 ± 4.61	<.0001
Week 9	46.23 ± 41.52	4.57 ± 3.94	<.0001
Week 12	57.68 ± 46.54	5.48 ± 3.19	<.0001
P value (Week 6 *vs* Baseline)	<.0001	<.0001	
P value (Week 9 *vs* Baseline)	<.0001	<.0001	
P value (Week 12 *vs* Baseline)	<.0001	<.0001	
Catechins (ng/ml)			
Baseline	0.20 ± 0.67	0.17 ± 0.64	-
Week 6	3.06 ± 2.25	1.48 ± 1.94	0.0021
Week 9	2.26 ± 1.55	1.26 ± 2.11	0.0392
Week 12	2.14 ± 1.22	0.88 ± 0.52	<.0001
P value (Week 6 *vs* Baseline)	<.0001	0.0002	
P value (Week 9 *vs* Baseline)	<.0001	0.0032	
P value (Week 12 *vs* Baseline)	<.0001	<.0001	

Values represent means ± SD

Table S11. Plasma methylxantine concentrations after daily consumption of 30 g chocolate for 6, 9 and 12 weeks

	High-flavanol chocolate (n=33)	Low-flavanol chocolate (n=41)	*P* value of changes between treatments
Theobromine (ng/ml)			
Baseline	232.1 ± 333.5	222.3 ± 276.5	-
Week 6	3,831.7 ± 1,101.7	3,529.6 ± 1,073.9	0.66
Week 9	3,029.1 ± 1032.6	2,528.1 ± 888.7	0.18
Week 12	2,511.8 ± 1,080.6	2,166.2 ± 776.7	0.32
P value (Week 6 *vs* Baseline)	<.0001	<.0001	
P value (Week 9 *vs* Baseline)	<.0001	<.0001	
P value (Week 12 *vs* Baseline)	<.0001	<.0001	
Theophylline (ng/ml)			
Baseline	192.0 ± 179.5	195.1 ± 223.3	-
Week 6	487.9 ± 306.4	450.8 ± 384.5	0.78
Week 9	407.9 ± 280.4	386.8 ± 298.2	0.91
Week 12	330.7 ± 205.7	308.7 ± 216.6	0.78
P value (Week 6 *vs* Baseline)	<.0001	0.0003	
P value (Week 9 *vs* Baseline)	<.0001	0.0010	
P value (Week 12 *vs* Baseline)	0.0058	0.0139	
Caffeine (ng/ml)			
Baseline	360.4 ± 574.9	355.2 ± 586.4	-
Week 6	2,083.7 ± 2,312.4	1,379.8 ± 1,412.1	0.11
Week 9	1,403.3 ± 1,283.4	1,190.1 ± 1,230.1	0.42
Week 12	1,493.0 ± 1,391.8	1,005.1 ± 928.0	0.11
P value (Week 6 *vs* Baseline)	<.0001	0.0004	
P value (Week 9 *vs* Baseline)	<.0001	0.0002	
P value (Week 12 *vs* Baseline)	0.0002	0.0012	

Values represent means ± SD

Table S12. Dietary polyphenols* after daily consumption of 30 g chocolate for 6, 9 and 12 weeks

	High-flavanol chocolate (n=33)	Low-flavanol chocolate (n=41)	*P* value of changes between treatments
Epicatechins (mg/day)*			
Baseline	10.50 ± 7.54	10.65 ± 7.00	-
Week 6	9.02 ± 6.95	9.10 ± 6.57	0.51
Week 9	6.52 ± 5.66	7.2 ± 4.66	0.67
Week 12	5.66 ± 5.09	8.10 ± 5.61	0.48
P value (Week 6 *vs* Baseline)	0.44	0.08	
P value (Week 9 *vs* Baseline)	0.0082	0.0022	
P value (Week 12 *vs* Baseline)	0.0006	0.06	
Catechins (mg/day)*			
Baseline	11.94 ± 7.91	14.35 ± 9.28	-
Week 6	9.10 ± 7.16	11.11 ± 8.27	0.37
Week 9	7.52 ± 5.30	9.76 ± 7.40	0.29
Week 12	7.75 ± 5.03	9.05 ± 7.03	0.39
P value (Week 6 *vs* Baseline)	0.23	0.02	
P value (Week 9 *vs* Baseline)	0.0025	0.0015	
P value (Week 12 *vs* Baseline)	0.01	0.02	

*** P**olyphenol consumption was calculated by FFQ

Values represent means ± SD

Table S13. Skin elasticity parameters after 3-week washout

	High-flavanol chocolate (n=33)	Low-flavanol chocolate (n=41)	*P* value of changes between treatments
Skin elasticity parameters (mm)			
**Skin distensibility**:			
**Arm**			
Week 12	0.21 ± 0.06	0.19 ± 0.07	-
**Week 15**	0.19 ± 0.06	0.18 ± 0.07	**0.04**
P value (Week 15 *vs* Week 12)	**0.0032**	0.44	
Temple			
Week 12	0.20 ± 0.06	0.20 ± 0.06	-
Week 15	0.19 ± 0.06	0.19 ± 0.07	0.89
P value (Week 15 *vs* Week 12)	0.09	0.22	
Overall elasticity:			
Arm			
Week 12	0.76 ± 0.10	0.75 ± 0.08	-
Week 15	0.76 ± 0.09	0.76 ± 0.08	0.50
P value (Week 15 *vs* Week 12)	0.82	0.46	
Temple			
Week 12	0.59 ± 0.12	0.57 ± 0.11	-
Week 15	0.58 ± 0.12	0.57 ± 0.10	0.37
P value (Week 15 *vs* Week 12)	0.14	0.95	
Net elasticity:			
Arm			
Week 12	0.81 ± 0.22	0.80 ± 0.23	-
Week 15	0.81 ± 0.26	0.81 ± 0.23	0.91
P value (Week 15 *vs* Week 12)	0.86	0.73	
Temple			
Week 12	0.52 ± 0.19	0.48 ± 0.12	-
Week 15	0.51 ± 0.17	0.51 ± 0.17	0.05
P value (Week 15 *vs* Week 12)	0.45	0.04	

Values represent means ± SD

Table S14. Plasma polyphenol concentrations after 3-week washout

	High-flavanol chocolate (n=33)	Low-flavanol chocolate (n=41)	*P* value of changes between treatments
Epicatechins (ng/ml)			
Week 12	57.68 ± 46.54	5.48 ± 3.19	<.0001
Week 15	0.69 ± 0.58	0.61 ± 0.48	<.0001
P value (Week 15 *vs* Week 12)	<.0001	<.0001	
Catechins (ng/ml)			
Week 12	2.14 ± 1.22	0.88 ± 0.52	<.0001
Week 15	0.89 ± 0.80	0.87 ± 0.67	0.0005
P value (Week 15 *vs* Week 12)	0.0002	0.96	

Values represent means ± SD

Table S15. Plasma methylxantine concentrations after 3-week washout

	High-flavanol chocolate (n=33)	Low-flavanol chocolate (n=41)	*P* value of changes between treatments
Theobromine (ng/ml)			
Week 12	2,511.8 ± 1,080.6	2,166.2 ± 776.7	-
Week 15	229.8 ± 254.7	202.3 ± 246.9	0.07
P value (Week 15 *vs* Week 12)	<.0001	<.0001	
Theophylline (ng/ml)			
Week 12	330.7 ± 205.7	308.7 ± 216.6	-
Week 15	190.0 ± 146.0	182.8 ± 140.0	0.69
P value (Week 15 *vs* Week 12)	<.0001	0.0008	
Caffeine (ng/ml)			
Week 12	1,493.0 ± 1,391.8	1,005.1 ± 928.0	-
Week 15	312.2 ± 253.8	315.7 ± 346.9	0.10
P value (Week 15 *vs* Week 12)	<.0001	<.0001	

Values represent means ± SD

Table S16. Skin hydration after 3-week washout

	High-flavanol chocolate (n=33)	Low-flavanol chocolate (n=41)	*P* value of changes between treatments
Skin hydration (units**)			
Arm			
Week 12	31.04 ± 8.54	32.51 ± 7.54	-
Week 15	32.86 ± 7.29	33.38 ± 7.17	0.76
P value (Week 15 *vs* Week 12)	0.11	0.39	
Temple			
Week 12	39.61 ± 10.31	39.27 ± 9.94	-
Week 15	40.79 ± 11.96	40.91 ± 9.12	0.96
P value (Week 15 *vs* Week 12)	0.37	0.23	

** Arbitrary Corneometer® units
Values represent means ± SD

Table S17. Dietary polyphenol consumption* after 3-week washout

	High-flavanol chocolate (n=33)	Low-flavanol chocolate (n=41)	*P* value of changes between treatments
Epicatechins (mg/day) *			
Week 12	5.66 ± 5.09	8.10 ± 5.61	-
Week 15	6.59 ± 5.47	7.27 ± 4.75	0.12
P value (Week 15 *vs* Week 12)	0.24	0.31	
Catechins (mg/day)*			
Week 12	7.75 ± 5.03	9.05 ± 7.03	-
Week 15	8.02 ± 6.64	8.49 ± 4.17	0.52
P value (Week 15 *vs* Week 12)	0.68	0.61	

* Polyphenol consumption was calculated by FFQ
Values represent means ± SD

Figure S1. Graph depicting immediate deformation or skin extensibility (Ue), delayed distention (Uv), final deformation (Uf) and immediate retraction (Ur)

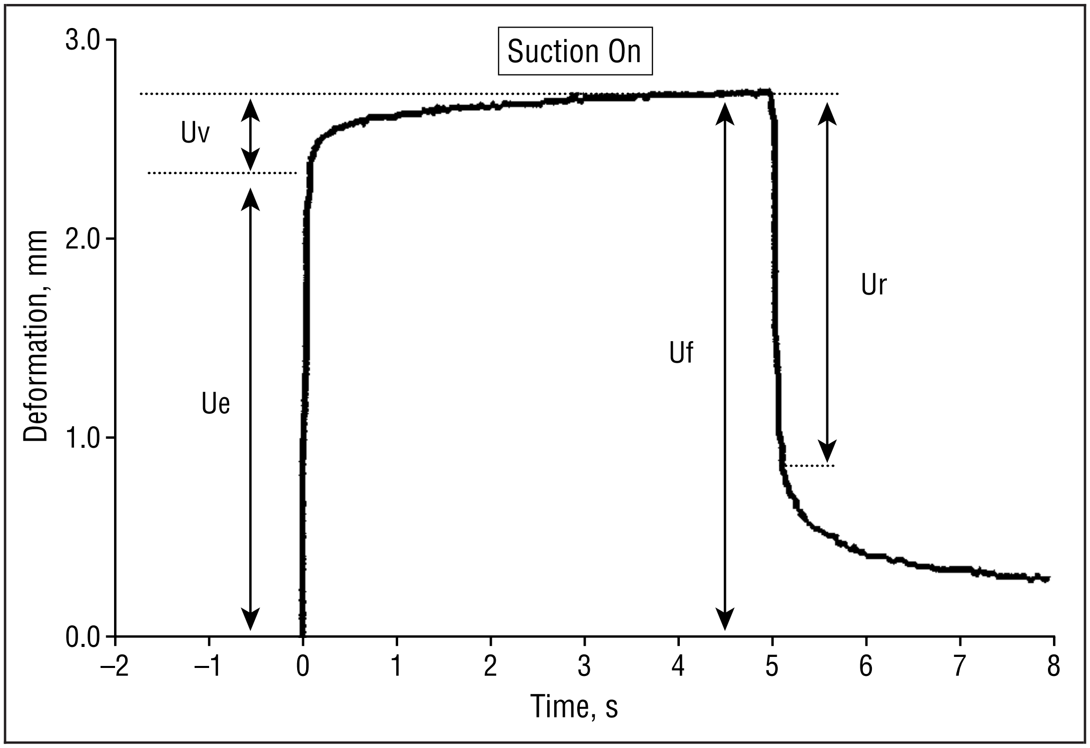

Figure S2. Changes in MED at 12 weeks by month of primary endpoint evaluation

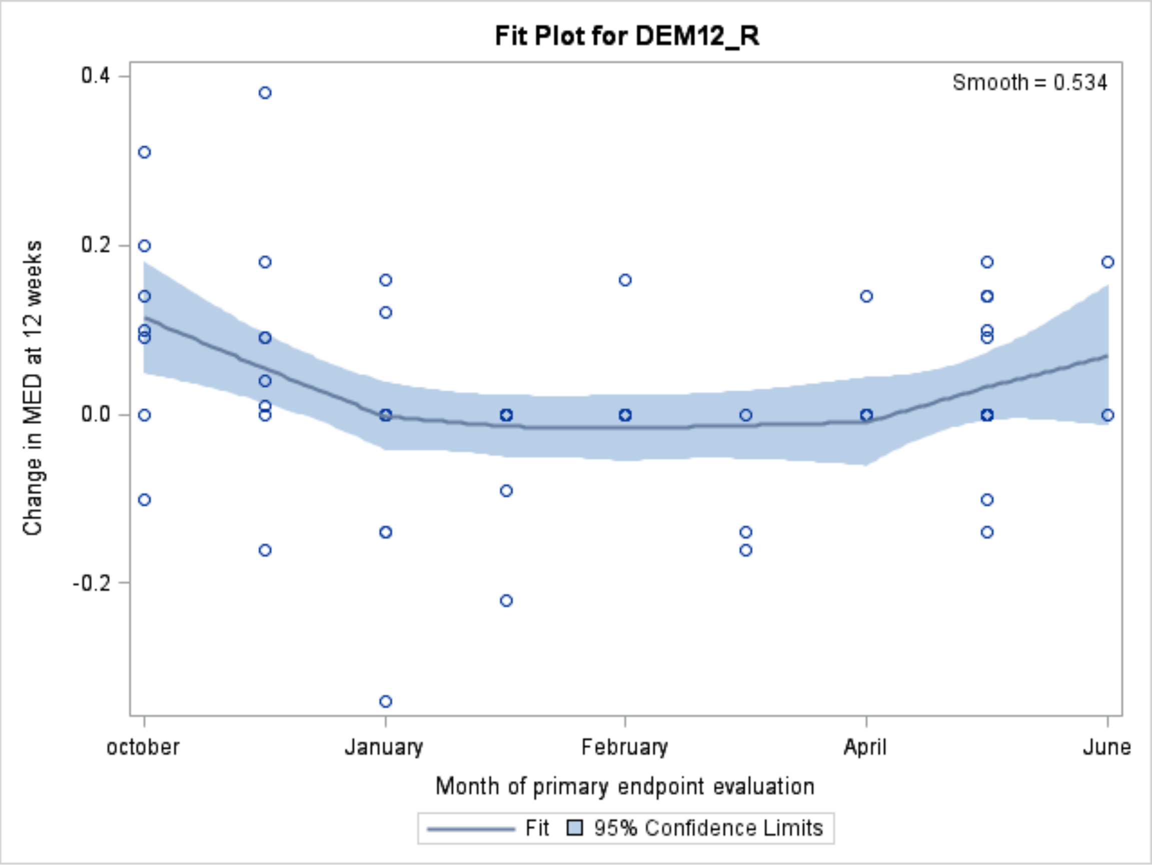

Supplement: Additional file 1 — Chocolate flavanols and skin photoprotection: a parallel, double-blind, randomized clinical trial. [file 1475-2891-13-66-S1.pdf]
